# Maltreatment in childhood and intimate partner violence: A latent class growth analysis in a South African pregnancy cohort

**DOI:** 10.1016/j.chiabu.2018.08.020

**Published:** 2018-12

**Authors:** Whitney Barnett, Sarah Halligan, Jon Heron, Abigail Fraser, Nastassja Koen, Heather J. Zar, Kirsty A. Donald, Dan J. Stein

**Affiliations:** a Department of Paediatrics and Child Health, Red Cross War Memorial Children’s Hospital, Medical Research Council Unit on Child & Adolescent Health, University of Cape Town, South Africa; b Department of Psychology, Bath University, United Kingdom; c Population Health Sciences, Bristol Medical School, University of Bristol, United Kingdom; d MRC Integrative Epidemiology Unit at the University of Bristol, Bristol, United Kingdom; e Department of Psychiatry and Mental Health, University of Cape Town, South Africa; f South African Medical Research Council Unit on Risk and Resilience in Mental Disorders, Cape Town, South Africa; g Division of Developmental Paediatrics, Department of Paediatrics & Child Health, Red Cross War Memorial Children’s Hospital, University of Cape Town, Cape Town, South Africa

**Keywords:** Intimate partner violence, Childhood maltreatment, Latent class, Intergenerational trauma

## Abstract

Intimate partner violence (IPV) is a significant global problem, prevalent in low and middle-income countries (LMICs). IPV is particularly problematic during the perinatal and early postnatal period, where it is linked with negative maternal and child health outcomes. There has been little examination of profiles of IPV and early life adversity in LMIC contexts. We aimed to characterize longitudinal IPV and to investigate maternal maltreatment in childhood as a predictor of IPV exposure during pregnancy and postnatally in a low resource setting. This study was nested in the Drakenstein Child Health Study, a longitudinal birth cohort. Maternal IPV (emotional, physical and sexual) was measured at six timepoints from pregnancy to two years postpartum (n = 832); sociodemographic variables and maternal maltreatment in childhood were measured antenatally at 28–32 weeks’ gestation. Associations between maternal maltreatment in childhood and IPV latent class membership (to identify patterns of maternal IPV exposure) were estimated using multinomial and logistic regression. We observed high levels of maternal maltreatment during childhood (34%) and IPV during pregnancy (33%). In latent class analysis separating by IPV sub-type, two latent classes of no/low and moderate sexual IPV and three classes of low, moderate, and high emotional and physical IPV (separately) were detected. In combined latent class analysis, including all IPV sub-types together, a low, moderate and high exposure class emerged as well as a high antenatal/decreasing postnatal class. Moderate and high classes for all IPV sub-types and combined analysis showed stable intensity profiles. Maternal childhood sexual abuse, physical abuse and neglect, and emotional abuse predicted membership in high IPV classes, across all domains of IPV (aORs between 1.99 and 5.86). Maternal maltreatment in childhood was associated with increased probability of experiencing high or moderate intensity IPV during and around pregnancy; emotional neglect was associated with decreasing IPV class for combined model. Intervening early to disrupt this cycle of abuse is critical to two generations.

## 1 Introduction

The World Health Organisation (WHO) found lifetimeprevalence rates of exposure to physical or sexual partner violence of 15%–71% in a recent multi-country study (Abramsky et al., 2011). The epidemic of intimate partner violence (IPV), predominantly affecting women, is particularly high in low and middle-income countries (LMICs), including South Africa (Dunkle, Jewkes, Brown, Gray et al., 2004; Dunkle, Jewkes, Brown, Yoshihama et al., 2004; Jewkes et al., 2008). Levels of violence in South Africa are some of the highest globally; the intimate female homicide rate in South Africa is 5.6 per 100,000, more than double the rate in the United States (Mathews et al., 2004). This amounts to one woman being killed by an intimate partner every 8 hours (Abrahams, Matthews, Jewkes, Martin, & Lombard, 2012). In South Africa interpersonal violence, including IPV, accounts for 10.9% of all disability-adjusted life years (DALYs) (Groves et al., 2015).

Of particular concern is violence toward pregnant women, as it carries unique pregnancy-related risk and can have long-term consequences for both maternal and child health. A review of African studies focussing on IPV during pregnancy, found that prevalence in African countries is among the highest globally, with reported rates from 2% (Nigeria) to 57% (Uganda), (Shamu, Abrahams, Temmerman, Musekiwa, & Zarowsky, 2011). IPV exposure has been linked to physical and psychological stress, anxiety, low energy, diminished social function and substance use disorders (Campbell, 2002; Duvvury, Callan, Carney, & Raghavendra, 2013) all of which may impact foetal growth, development and neurocognitive outcomes in the children of exposed women (Gilbert, Bauer, Carroll, & Downs, 2013; Gustafsson, Coffman, & Cox, 2015; Udo, Sharps, Bronner, & Hossain, 2016). Further, IPV exposure during pregnancy has been linked to increased risk of pregnancy loss (Huth-Bocks, Levendosky, & Bogat, 2002), preterm labour, pregnancy complications and delivering low birth weight infants (Bronte-Tinkew, Zaslow, Capps, Horowitz, & McNamara, 2007; Campbell, 2002; Valladares, Ellsberg, Pena, Hogberg, & Persson, 2002). In the South African context, IPV also increases risk of maternal – and hence vertical - HIV infection (Jewkes, Dunkle, Koss et al., 2006; Jewkes, Dunkle, Nduna et al., 2006; Li et al., 2014; Maman et al., 2002).

Few studies have investigated longitudinal patterns of IPV during pregnancy and the postpartum period. The limited existing evidence is inconsistent, with some studies showing an increased risk during pregnancy compared to postpartum (Mahenge, Stöckl, Abubakari, Mbwambo, & Jahn, 2016) and others showing a decreased risk from pre-pregnancy to pregnancy (Fawole, Hunyinbo, & Fawole, 2008; Saltzman, Johnson, Gilbert, & Goodwin, 2003). Where studies have found that pregnancy increases a woman’s risk for IPV victimisation, reasons for this have included increased financial strain (Bacchus, Mezey, & Bewley, 2006; Xu et al., 2005) or conflict arising from an unwanted pregnancy (Fanslow, Silva, Robinson, & Whitehead, 2008). Investigating longitudinal IPV exposure perinatally may provide insight into how pregnancy affects the pattern of maternal risk of IPV exposure over the course of this critical period. Further, few studies have looked at the intensity of IPV in women during this time of increased vulnerability.

Physical, sexual and emotional abuse or neglect during childhood is widespread in South Africa, with reported prevalence rates of 9% to 34% (Burton & Artz, 2015; Meinck, Cluver, & Boyes, 2015). Studies in high-income settings have found links between physical abuse as well as sexual abuse in childhood and risk of IPV victimisation (Yan & Karatzias, 2016) or perpetration in adulthood (McKinney, Caetano, Ramisetty-Mikler, & Nelson, 2009). In particular, childhood sexual abuse has been well documented as a predictor of sexual revictimisation (Classen, Palesh, & Aggarwal, 2005). There are limited data from African settings; these show that physical or sexual childhood abuse increases the risk of IPV in adulthood (Abramsky et al., 2011). In addition, South African studies have shown a link between male childhood abuse and IPV perpetration (Abrahams & Jewkes, 2005; Jewkes, Dunkle, Koss et al., 2006; Jewkes, Dunkle, Nduna et al., 2006) and an increased risk of IPV victimisation where men or women had a history of childhood sexual abuse (Dunkle, Jewkes, Brown, Gray et al., 2004; Dunkle, Jewkes, Brown, Yoshihama et al., 2004; Daigneault, Hebert, & McDuff, 2009). However, none of these studies investigated childhood emotional neglect or abuse, which typically occurs alongside other forms of abuse, and has a unique and sometimes compounding affect above physical or sexual IPV, in particular for mental health outcomes (Ludermir, Lewis, Valongueiro, de Araujo, & Araya, 2010; Shamu, Zarowsky, Roelens, Temmerman, & Abrahams, 2016; Tiwari et al., 2008).

The current study aimed to address critical gaps in the literature relevant to settings where high rates of childhood maltreatment and IPV co-occur. First, it investigated IPV exposure longitudinally, from mid-pregnancy through to 2 years following birth, to better understand patterns of risk for pregnant women. Second, it investigated the association of maternal maltreatment in childhood with perinatal and subsequent IPV, and the possibility of differential associations across sub-types of both childhood maltreatment and adult IPV exposure. Importantly, data for this study were derived from a pregnancy cohort from a low resource setting in South Africa, where high rates of childhood maltreatment and IPV co-exist, and where unique cultural and social factors may impact associations differently than in high-income country settings.

## 2 Methods

This study is nested in the Drakenstein Child Health Study (DCHS), a multidisciplinary birth cohort investigating the determinants of child health in a peri-urban area in South Africa (Stein et al., 2015; Zar, Barnett, Myer, Stein, & Nicol, 2015). Data used in the current study were collected from pregnant women enrolled into the DCHS from March 2012 to March 2015.

### 2.1 Setting

The DCHS is located in the Drakenstein area in the town of Paarl, a peri-urban area, 60 km outside Cape Town, South Africa with a population of approximately 200,000. More than 90% of the population access health care in the public sector including antenatal and child health services. This area has a well-established, free primary health care system. An area of focus in the DCHS is investigating maternal psychosocial risk factors of child health (Stein et al., 2015).

### 2.2 Participants

Pregnant women were recruited from two primary health care clinics, Mbekweni (serving a predominantly black African community) and TC Newman (serving a mixed ancestry community). Mothers were enrolled in the DCHS at 20 to 28 weeks’ gestation while attending routine antenatal care and are prospectively followed through their pregnancy until 5 years postnatally. Women were eligible for the study if they were 18 years or older, between 20–28 weeks gestation, planned attendance at one of the two recruitment clinics and intended to remain in the area. Data included in the current study were collected antenatally at 28–32 weeks’ gestation and postnatally at 10 weeks, 6, 12, 18 and 24 months.

Between March 2012 and March 2015, 1225 pregnant women were enrolled into the DCHS antenatally; 88 (7.2%) mothers were lost to follow up antenatally, had a miscarriage or a stillbirth. Of the 1137 women who had live births, 100 mothers did not attend the second antenatal visit, where sociodemographic variables and childhood maltreatment data were collected. Of the 1037 mothers who did attend this visit, 832 (80%) were included in this analysis, restricted to those who contributed data for at least 3 of the 6 time points. A sensitivity analysis was done to compare all included variables between mothers included and excluded in the current analysis (Supplemental Table 1, further detail in Statistical Analysis section).

### 2.3 Measures

IPV exposure: The Intimate Partner Violence Questionnaire (IPVQ) is a 12-item inventory adapted from the WHO multicountry study (Jewkes, 2002) and the Women’s Health Study in Zimbabwe (Shamu et al., 2011) and assessed recent (past-year) exposure to emotional (4 of 12 questionnaire items), physical (5 of 12 items), and sexual abuse (3 of 12 items). Mothers were asked about exposure to partner behavior and frequency of occurrence (“never”, “once”, “a few times” or “many times”). Mothers completed the IPVQ at the 28–32 week antenatal visit and at 10 weeks, 6, 12, 18 and 24 months postpartum. Partner behavior indicating emotional IPV included having been insulted or made to feel bad, having been humiliated in front of others, intentionally scared or intimidated or threatened with physical harm. Physical IPV included being slapped, pushed, shoved, hit with an object, beaten or choked. Sexual IPV exposure was classified based on having been forced to have sex, afraid not to have sex or forced to do something sexual which was degrading or humiliating. Using questionnaire responses mothers were grouped into four categories of exposure: no IPV where all past year behaviours were “never” experienced; isolated or low IPV was designated where any past year behaviours were experienced as “once” and none more frequently than once; moderate where past year behavior was experienced “a few times”; and high where “many times” was indicated. This was done at each of the six time points to investigate changing exposure patterns during the 2 year period of follow up. Scoring guidelines were devised for the purposes of this study, and were based on prior work in South Africa (Dunkle, Jewkes, Brown, Gray et al., 2004; Dunkle, Jewkes, Brown, Yoshihama et al., 2004).

Maternal Maltreatment in Childhood: The Childhood Trauma Questionnaire (CTQ) (Bernstein et al., 1994) is a 28- item inventory assessing three domains of childhood abuse (sexual, physical, and emotional), and two domains of childhood neglect (physical and emotional), occurring at or before the age of 12 years. Each item is scored on a frequency scale from 1 (“never true”) to 5 (“very often true”), such that each subscale (domain of abuse or neglect) is scored on a spectrum from 5 (no history of abuse or neglect) to 25 (very extreme history of abuse or neglect). Dichotomous variables were included in the present analysis, as previously described, such that above threshold for each domain was defined as: physical neglect (score of ≥8); physical abuse (score of ≥8); emotional neglect (score of ≥10); emotional abuse (score of ≥9); and sexual abuse (score of ≥6) (Bernstein et al., 1994). Mothers completed the CTQ antenatally at 28–32 weeks’ gestation.

Sociodemographic variables were collected from an adapted questionnaire used in the South African Stress and Health (SASH) study (Myer, Stein, Grimsrud, Seedat, & Williams, 2008). Maternal age, income [<R,1000/month (100USD) or > R1,000/month], education (any secondary versus completed secondary), employment and partnership status (single or married/marriage-like relationship) were self-reported antenatally at 28–32 weeks’ gestation.

### 2.4 Ethical considerations

The DCHS was approved by the Faculty of Health Sciences, Human Research Ethics Committee, University of Cape Town (401/2009) and by the Western Cape Provincial Health Research committee. Mothers provided informed consent in their preferred language: English, Afrikaans or isiXhosa and were given R100 (approximately 8USD) for travel reimbursement to reach study sites. Study staff were trained on the content of questionnaires and ethical conduct of violence research, including confidentiality and safety issues. Interviews were conducted privately, data were de-identified and only accessible by study staff to ensure confidentiality. Staff were trained to recognise signs of mental health issues (depression, PTSD symptoms and suicide risk) as well as circumstances endangering mothers or children, including Department of Health mandatory reporting requirements for endangerment. Where identified, staff were trained to refer participants to appropriate care or social services in the Paarl area specialising in the issue identified (including support services for IPV, substance abuse and mental health issues). Further, all women involved in the study, independent of identified mental or physical health issues, receive information regarding social and support service providers in the area.

## 3 Statistical analysis

Latent class growth analysis (LCGA) was used to derive latent classes indicating severity patterns of emotional, physical and sexual recent IPV victimisation separately and across the six time points included (pregnancy, 10 weeks, 6, 12, 18, 24 months). LCGA is a type of growth mixture modeling (GMM) in which there is no within-class variability modelled. Categorical IPV data (consisting of 4 categories of exposure: no, low, moderate and high) was used to estimate the latent classes. Compared to latent class analysis (LCA), LCGA of categorical data allows for a more parsimonious model. In the Mplus implementation, continuous Gaussian variables are assumed to underpin each categorical class-indicator, so the item thresholds are also modelled more efficiently. LCGA was used to create longitudinal classes separately of IPV sub-types (Galatzer-Levy, 2015).

A second analysis was done to investigate patterns across all sub-types of IPV. This analysis builds upon the association models discussed above – by utilising latent class analysis (LCA) to determine combined latent classes for all IPV sub-types (across same 6 time points) to investigate differential profiles considering all IPV sub-types (sexual, physical & emotional). LCA was used as an analytic approach to allow within class variation to enable different patterns between groups of key variables to emerge within each class; thus allowing cross sectional and longitudinal heterogeneity to be captured. Both LCGA and LCA group individuals into classes based on profiles of indicator variables, allowing identification of heterogenous groups of homogenous person-centred patterns (Muthen & Muthen, 2000).

LCGA and LCA analyses were completed in MPlus 8.0. Optimal number of classes were determined based on multiple statistical criteria, including Akaike Information Criterion (AIC) and sample size adjusted Bayesian Information Criterion (ssaBIC) as well as sufficient class size (Muthen & Muthen, 2000). To ensure a meaningful class size and allow clinically relevant interpretation, we excluded any models where the smallest class was fewer than 30 women, similar to other studies (Meyer, Yu, Hermosilla, & Stark, 2017; Olson, Choe, & Sameroff, 2017; Solomon et al., 2018). To investigate associations of maternal maltreatment in childhood (by subtype) with LCGA and LCA class membership for IPV, we utilised the bias-adjusted 3-step approach, which takes into account inaccuracy of class assignment (Asparouhov & Muthén, 2014; Feingold, Tiberio, & Capaldi, 2014). Using this 3-step approach, multinomial logistic regression analyses were performed; sociodemographic variables (education, employment, site of enrolment, income, maternal age and whether married) were included in the LCGA and LCA models as covariates.

All analyses were restricted to mothers who contributed data for at least three of the six time points to enable investigation of changes over time for all included women [832 (73.2%) of the 1137 women who had live births, were included]. A sensitivity analysis was done to ensure there were no meaningful differences for key variables (childhood maltreatment, antenatal IPV or sociodemographics) for mothers included compared to mothers excluded in the current analysis (Supplemental Table 1). No meaningful differences were found for variables included in the present analysis.

## 4 Results

### 4.1 Sociodemographic, maternal maltreatment in childhood and IPV variables

The study sample was characterised by low levels of education, and a minority of mothers were employed (25%), or were married/in a stable relationship (40%). The majority were born in the study area (Paarl; 64%), and earned >R1,000 per month (USD100). Median age was 26.2 years (Table 1). Levels of childhood exposure to maltreatment were high, with over a third of the sample reporting at least one form of maltreatment and 5% reporting all five types (Table 2). Polyvictimisation based on IPV class membership was prevalent; 44% of women were grouped into high or moderate IPV classes for at least one sub-type with 6% grouped into high or moderate classes for all three IPV sub-types, Table 2. IPV severity prevalence rates across time points and by sub-type are presented in Supplemental Table 2.

**Table 1:** Sociodemographic characteristics of study population by latent class membership (n = 832).

Variable	Total sample	Emotional IPV (high exposure class)	Physical IPV (high exposure class)	Sexual IPV (high exposure class)
	n = 832	n = 37	n = 69	n = 58
*Demographic variables*^a^				
TC Newman	389 (47%)	27 (73%)	47 (68%)	48 (83%)
Maternal education				
Some secondary	531 (64%)	28 (76%)	52 (75%)	45 (78%)
Completed secondary	301 (36%)	9 (24%)	17 (24%)	13 (22%)
Maternal Birthplace				
Outside Paarl	302 (36%)	4 (11%)	9 (13%)	0 (0%)
Paarl	530 (64%)	33 (89%)	60 (87%)	58 (100%)
Maternal Employment				
Unemployed	628 (75%)	30 (81%)	61 (88%)	46 (79%)
Working	204 (25%)	7 (19%)	8 (12%)	12 (21%)
Partnership status				
Single	496 (60%)	18 (49%)	41 (59%)	36 (62%)
Married/marriage-like	336 (40%)	19 (51%)	28 (41%)	22 (38%)
Income				
<R1,000/mo	326 (39%)	13 (35%)	29 (42%)	303 (39%)
>R1,000/mo	506 (61%)	24 (65%)	40 (58%)	471 (61%)
Maternal age	26.2 (22.1, 30.9)	27.0 (23.6, 33.0)	24.7 (21.9, 29.2)	26.1 (22.2, 29.9)

a demographic variables were collected antenatally at 28–32weeks’ gestation.

**Table 2:** Childhood maltreatment prevalence and polyvictimisation in study population (n = 832).

Variable	Total sample	Emotional IPV (high exposure class)	Physical IPV (high exposure class)	Sexual IPV (high exposure class)	
	n=832	n=37	n=69	n=58	
*Psychosocial variables*					
Childhood Maltreatment (above threshold)^a^					
Physical Neglect	279 (34%)	18 (49%)	32 (46%)	31 (53%)	
Emotional Abuse	263 (32%)	23 (62%)	39 (57%)	41 (71%)	
Emotional Neglect	230 (28%)	22 (59%)	34 (49%)	35 (60%)	
Physical Abuse	189 (23%)	15 (41%)	29 (42%)	22 (38%)	
Sexual Abuse	137 (16%)	13 (35%)	26 (38%)	25 (43%)	
Any maltreatment	284 (34%)	24 (65%)	46 (67%)	42 (72%)	
Polyvictimisation: childhood maltreatment					
2 types	138 (17%)				
3 types	80 (10%)				
4 types	46 (6%)				
5 types	39 (5%)				
Polyvictimisation: Intimate Partner Violence (overlapping class membership)^b^					
None	465 (56%)				
1 type	142 (17%)				
2 types	173 (21%)				
3 types	52 (6%)				

a childhood maltreatment variables were collected antenatally at 28–32 weeks’ gestation.

b number and percentage of women exposed to high or moderate IPV for emotional, physical or sexual IPV classes.

### 4.2 Emotional IPV latent class analysis and association with child maltreatment

**Fig. 1:**
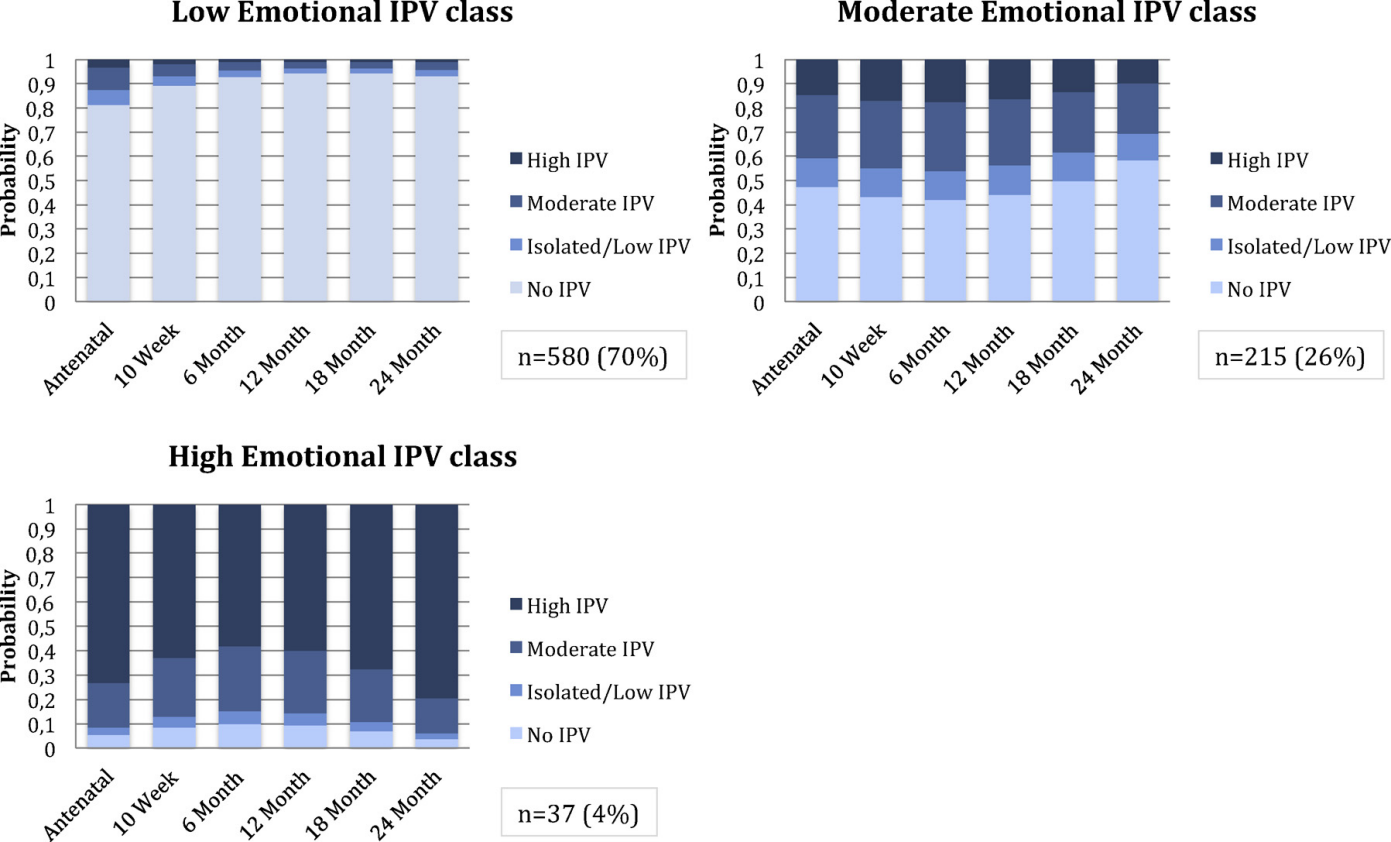
Emotional Intimate Partner Violence Latent classes: probabilities of exposure across visits.

Model fit statistics for models with a varying number of classes are presented in Table 3. A 3-class model was chosen for emotional IPV, based on lowest ssaBIC and AIC while retaining a meaningful class size; entropy was 0.732. Women in the first class were characterised by no or isolated/low emotional IPV over time with increased probability of emotional IPV exposure during pregnancy compared to postpartum (n = 580; 70%). Women in the second class were characterised by moderate emotional IPV over time (n = 215; 26%); those in the third class were characterised by high IPV across time points (n = 37; 4%), Fig. 1.

**Table 3:** Fit statistics for LCGA and LCA models for IPV^a^.

	# classes	ssaBIC	AIC	Entropy	Smallest class (%)
**Emotional**	**2**	5934.726	5920.792	0.779	171 (21)
	**3**	**5879.291**	**5859.165**	**0.732**	**37 (4)**
	**4**	5867.095	5840.775	0.78	12 (1)
	**5**	5872.415	5839.903	0.835	10 (1)
**Physical**	**2**	5617.393	5603.459	0.777	178 (21)
	**3**	**5578.992**	**5558.865**	**0.643**	**69 (8)**
	**4**	5569.026	5542.707	0.688	8 (1)
	**5**	5571.706	5539.194	0.728	8 (1)
**Sexual**	**2**	**2031.617**	**2017.684**	**0.883**	**58 (7)**
	**3**	2033.464	2013.337	0.909	11 (1)
**Combined IPV**	2	13221.576	13052.563	0.900	193 (23)
	3	12960.099	12705.804	0.863	98 (12)
	**4**	**12922.318**	**12582.743**	**0.879**	**67 (8)**
	5	12947.766	12522.909	0.871	63 (8)
	6	12991.713	12481.574	0.874	22 (3)

a AIC = Akaike’s information criterion; ssaBIC = sample-size adjusted Bayesian information criterion; Selected models appear in **bold**. All models included covariates. – IPV variables collected antenatally, at 10 weeks, 6, 12, 18 & 24 months postnatally.

The no/low emotional IPV class was treated as the reference class for multinomial regression. Exposure to childhood abuse of all types was associated with membership in the high emotional IPV exposure class (versus the low/no exposure) [sexual abuse (OR 4.28; 95% CI 1.48, 12.39); emotional abuse (OR 4.31; 95% CI 1.67,11.13); physical abuse (OR 3.16; 95% CI 1.22, 8.18), emotional neglect (OR 2.76; 95% CI 1.31, 6.73) as well as physical neglect (OR 3.74; 95% CI 1.48, 9.44)], Table 4. Childhood experience of physical abuse (OR 2.01; 95% CI, 1.10, 3.69) and physical neglect (OR 1.67; 95% CI, 1.03, 2.70) was associated with membership in the moderate emotional IPV class, Table 4.

**Table 4:** Maternal childhood maltreatment type and associations with maternal perinatal IPV latent class membership by IPV sub-type (n = 832).

	Childhood	Emotional IPV: High vs Low class				Emotional IPV: Moderate vs Low class			
	maltreatment type^a^	OR^b^	AOR^c^	95% CI	p-value	OR^V^	AOR^c^	95% CI	p-value
**Emotional IPV**	Sexual Abuse	5.85	4.28	1.48; 12.39	0.007	1.99	1.54	0.87; 1.00	0.135
	Emotional Neglect	3.30	2.76	1.31; 6.73	0.025	1.82	1.69	0.99; 2.86	0.050
	Emotional Abuse	6.62	4.31	1.67; 11.13	0.003	2.16	1.68	0.99; 2.84	0.053
	Physical Neglect	5.99	3.74	1.48; 9.44	0.005	2.24	1.67	1.03; 2.70	0.036
	Physical Abuse	4.37	3.16	1.22; 8.18	0.017	2.19	2.01	1.10; 3.69	0.024

	Childhood	Physical IPV: High vs Low class				Physical IPV: Moderate vs Low class			
	maltreatment type^a^	OR^b^	AOR^c^	95% CI	p-value	OR^b^	AOR^c^	95% CI	p-value
**Physical IPV**	Sexual Abuse	6.81	5.86	2.63; 13.08	<0.001	1.32	0.88	0.45; 1.71	0.706
	Emotional Neglect	3.28	3.30	1.63; 6.64	0.001	0.96	0.67	0.94; 1.30	0.238
	Emotional Abuse	4.20	2.89	1.33; 6.30	0.007	1.80	1.57	0.87; 2.81	0.134
	Physical Neglect	4.47	2.79	1.40; 5.56	0.004	1.45	1.20	0.70; 2.07	0.518
	Physical Abuse	5.20	4.95	2.10; 11.70	<0.001	1.51	1.24	0.59; 2.63	0.569

	Childhood	Sexual IPV: High vs Low class			
	maltreatment type^a^	OR^b^	AOR^c^	95% CI	p-value
**Sexual IPV**	Sexual Abuse	6.81	5.44	2.43; 12.19	<0.001
	Emotional Neglect	2.37	1.89	0.95; 3.79	0.070
	Emotional Abuse	5.15	3.31	1.60; 6.94	0.001
	Physical Neglect	7.09	4.38	2.04; 9.41	<0.001
	Physical Abuse	5.07	4.54	2.07; 9.94	<0.001

a Childhood maltreatment sub-types included as dichotomous exposure (data collected antenatally at 28–32 weeks’ gestation).

b Unadjusted multinomial logistic regression model. Odds ratios generated for each childhood maltreatment type and association with maternal membership in indicated IPV exposure class (high versus low and moderate versus low exposure over time). All childhood maltreatment types were included in separate models because of collinearity between maltreatment sub-types.

c Adjusted multinomial logistic regression models were controlled for key sociodemographic variables (maternal age, maternal income, maternal employment, maternal education, site of enrolment and partnership status, collected antenatally at 28–32 weeks’ gestation). All childhood maltreatment types were included in separate models because of collinearity between maltreatment sub-type.

### 4.3 Physical IPV latent class analysis and association with child maltreatment

**Fig. 2:**
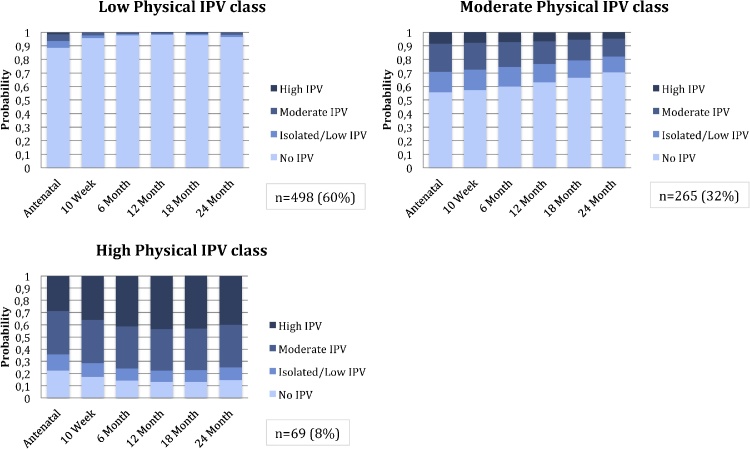
Physical Intimate Partner Violence Latent classes: probabilities of exposure across visits.

A 3-class model was chosen for physical IPV, based on lowest ssaBIC and AIC as well as meaningful class size; entropy was 0.643, Table 3. Women in the first class were characterised by low/isolated or no IPV with slightly increased probabilities of exposure during pregnancy, compared to postpartum (n = 498; 60%); women in the second class were characterised by moderate physical IPV over time (n = 265; 32%); and women in the third class were characterised by high IPV over time (n = 69; 8%), Fig. 2.

Exposure to each of the child maltreatment domains was significantly associated with membership in the high physical IPV class compared to the no/low physical IPV class. Exposure to child maltreatment increased this risk by between 2 to 5-fold [sexual abuse (OR 5.86; 95% CI 2.63, 13.08; p-value <0.001), emotional neglect (OR 3.30; 95% CI 1.63, 6.64), emotional abuse (OR 2.89; 95% CI 1.33, 6.30), physical neglect (OR 2.79; 95% CI 1.40, 5.56), physical abuse (OR 4.95; 95% CI 2.10, 11.70), Table 4. No domains of childhood exposure to maltreatment were significantly associated with membership in the moderate physical IPV class (versus the no/low physical IPV class).

### 4.4 Sexual IPV latent class analysis and association with child maltreatment

**Fig. 3:**
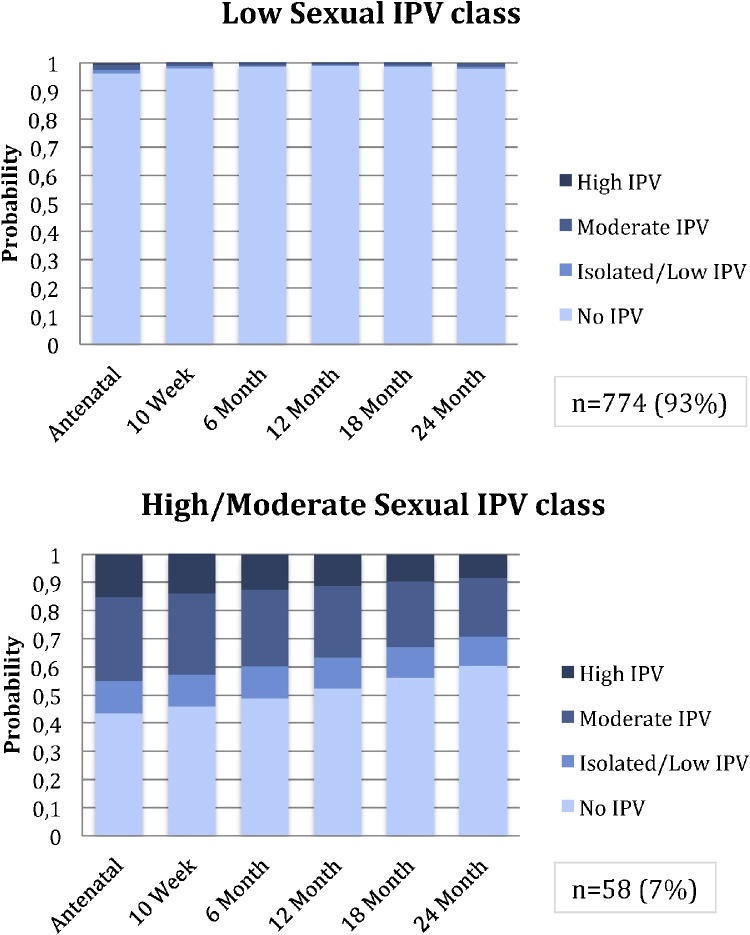
Sexual Intimate Partner Violence Latent classes: probabilities of exposure across visits.

For the LCA model of sexual IPV, the 2-class solution was chosen based on lowest ssaBIC and comparable AIC (to the 3-class solution) while retaining a meaningful class size; entropy was 0.879, indicating a good degree of class separation, Table 3. Women in the first class were characterised by low/no sexual IPV over time (n = 774;93%); women in the second class were characterised by high or moderate IPV over time (n = 58; 7%), Fig. 3.

Childhood exposure to sexual abuse (OR 5.44; 95% CI 2.43, 12.19), emotional abuse (OR 3.31; 95% CI 1.60, 6.94), physical neglect (OR 4.38; 95% CI 2.04, 9.41) and physical abuse (OR 4.54; 95% CI 2.07, 9.94) was associated with membership in the high/moderate sexual IPV class, Table 4.

### 4.5 Latent class analysis of maternal maltreatment in childhood and all IPV sub-types

**Fig. 4:**
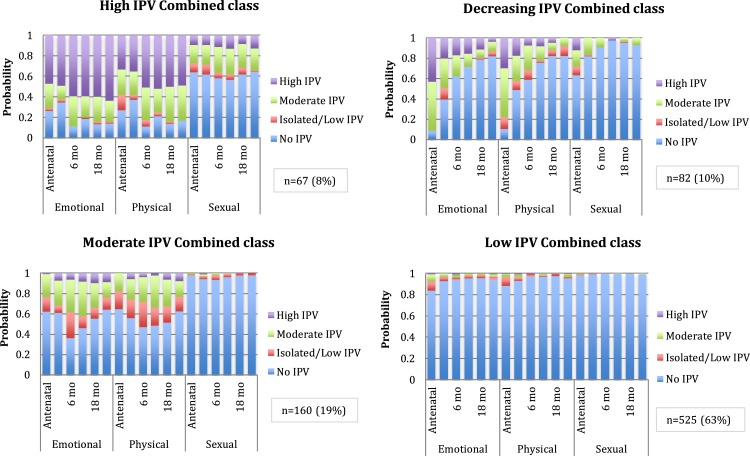
Latent class analysis of combined IPV sub-types (emotional, physical and sexual IPV).

To investigate profiles of maternal IPV across sub-types, latent class analysis was done. A four class model was chosen based on lowest ssaBIC (compared to the 3-class model), Table 5. Smallest class size was n = 67 (8%); entropy was 0.879. Class 1, high IPV combined class (n = 67), was characterised by high physical, emotional and sexual IPV from pregnancy to 2 years postnatally. Class 2, decreasing IPV combined class (n = 82), was characterised by high/moderate antenatal IPV across sub-types, with decreasing probability of IPV exposure through 2 years postnatally. Class 3, moderate IPV combined class (n = 160), was characterised by moderate emotional and physical IPV over time and very low sexual IPV. Class 4, low IPV combined class, (n = 525) had low/no probabilities of IPV across sub-types through 2 years postnatally, Fig. 4.

**Table 5:** Maternal childhood maltreatment type and associations with maternal perinatal IPV combined latent class membership (n = 832).

	Childhood maltreatment type^a^	OR^b^	AOR^c^	95%CI	p-value
		High vs Low class			
Combined IPV classes	Sexual Abuse	4.23	2.97	1.50; 5.91	0.002
	Emotional Neglect	2.45	1.99	1.11: 3.59	0.020
	Emotional Abuse	3.89	2.46	1.31; 4.61	0.004
	Physical Neglect	4.5	2.93	1.63; 5.31	<0.001
	Physical Abuse	4.71	3.61	1.85; 7.01	<0.001
		Moderate vs Low class			
	Sexual Abuse	1.48	1.33	0.79; 2.27	0.296
	Emotional Neglect	1.01	0.96	0.55; 1.66	0.890
	Emotional Abuse	1.75	1.64	0.99; 2.75	0.061
	Physical Neglect	1.36	1.16	0.73; 1.86	0.540
	Physical Abuse	2.06	2.23	1.26; 3.93	0.006
		Decreasing vs Low class			
	Sexual Abuse	2.37	1.93	0.94; 4.00	0.074
	Emotional Neglect	2.26	1.96	1.04; 3.66	0.037
	Emotional Abuse	2.33	1.75	0.92; 3.35	0.090
	Physical Neglect	1.36	1.16	0.73; 1.86	0.540
	Physical Abuse	2.02	1.46	0.66; 3.27	0.350

a Childhood maltreatment sub-types included as dichotomous exposure (data collected antenatally at 28–32 weeks’ gestation).

b Unadjusted multinomial logistic regression model. Odds ratios generated for each childhood maltreatment type and association with maternal membership in indicated IPV exposure class (high versus low, moderate versus low and decreasing versus low exposure over time). All childhood maltreatment types were included in separate models because of collinearity between maltreatment sub-types.

c Adjusted multinomial logistic regression models were controlled for key sociodemographic variables (maternal age, maternal income, maternal employment, maternal education, site of enrolment and partnership status, collected antenatally at 28–32 weeks’ gestation). All childhood maltreatment types were included in separate models because of collinearity between maltreatment sub-type.

Exposure to each of the child maltreatment domains was significantly associated with membership in the high combined IPV class (class 1) compared to the no/low IPV class (class 4). Exposure to child maltreatment increased this risk by between 2–3.6-fold [sexual abuse (aOR 2.97; 95% CI 1.50, 5.91), emotional neglect (aOR 1.99; 95% CI 1.11, 3.59), emotional abuse (OR 2.46; 95% CI 1.31, 4.61), physical neglect (aOR 2.93; 95% CI 1.63, 5.31), physical abuse (aOR 3.61; 95% CI 1.85, 7.01), Table 5. Maternal childhood exposure to physical abuse was significantly associated (aOR 2.23; 95%CI 1.26, 3.93) with membership in the moderate compared to low IPV class. Childhood emotional neglect was significantly associated (aOR 1.96; 95% CI 1.04, 3.66) with membership in the decreasing (versus low) IPV class.

## 5 Discussion

In this birth cohort study, based in a peri-urban area of South Africa, there were high reported levels of maternal maltreatment in childhood as well as IPV during pregnancy and during the postpartum period. The current study investigated longitudinal patterns of IPV severity, both separately by IPV sub-type (emotional, physical, sexual) and longitudinal patterns where all IPV sub-types were combined. In both analyses mothers were grouped into patterns of high, moderate and low exposure groups, indicating chronic exposure where present. However, also emerging in the combined analysis, was a group of mothers with very high levels of IPV antenatally – representing 10% of the sample, this is both a large proportion of women as well as women with exposure probabilities during pregnancy approximately as high as those in the high longitudinal exposure group. For both analyses considering longitudinal patterns of IPV, membership in the high versus low class was associated with all types of maternal childhood maltreatment, indicating a relationship between maternal childhood maltreatment and severity of adult exposure to IPV.

Previous South African studies have reported lower prevalence of IPV during pregnancy across sub-types [emotional at 15%–19.2% (Bernstein et al., 2016; Groves, Kagee, Maman, Moodley, & Rouse, 2012); physical at 4.7%–15% (Bernstein et al., 2016; Jewkes, Penn-Kekana, Levin, Ratsaka, & Schrieber, 2001); and sexual at 2%–3.2% (Bernstein et al., 2016; Groves et al., 2015)]. In our sample, prevalence of recent IPV during pregnancy was very high, with 24%, 18% and 6% of mothers reporting exposure for emotional, physical and sexual IPV respectively. This may be due to a number of factors. Few studies in South Africa have reported pregnancy rates of IPV, specifically by sub-type, though rates from our sample are within ranges where combined [30% for physical + sexual IPV (Dunkle, Jewkes, Brown, Gray et al., 2004; Dunkle, Jewkes, Brown, Yoshihama et al., 2004)] or reported from other African countries, such as Nigeria [39%, any-type IPV (Shamu et al., 2011)]. Further, of the two other South African studies reported, one was in Durban, which has lower overall rates of violence than the Western Cape, where the current study is located. The other was restricted to a sample of Black African participants, whereas reported rates were slightly higher in the mixed-ancestry community included in our study sample. Culture and gender norms within focal communities may in part explain our high prevalence of IPV. Zembe and colleagues investigated social risk factors and relationship power inequity and IPV in a sample in the Western Cape, finding high acceptability of violence in intimate relationships as well as gender norms supporting aggressive masculinity and subservient femininity, particularly in historically marginalised groups such as those included in the present study (Zembe, Townsend, Thorson, Silberschmidt, & Ekstrom, 2015).

Distinctive patterns of longitudinal IPV exposure emerged, when investigating separate IPV sub-type profiles, classifying women into high, moderate and low exposure groups over time for emotional and physical IPV and a high/moderate and no/low exposure group for sexual IPV. As expected the largest classes for each IPV sub-type were characterised by no/low IPV exposure. However a significant proportion of women were in high or moderate classes and, importantly, in low exposure groups there was an increase in IPV exposure during pregnancy across all IPV sub-types. Childhood physical, emotional and sexual abuse, as well as physical neglect, were all significantly associated with membership in the high IPV exposure classes compared to no or low exposure classes.

There was a large degree of longitudinal stability within classes, which indicates a consistent and repeated pattern of IPV exposure, based on IPV sub-type and intensity of exposure, from pregnancy through two years postpartum. Further, a large proportion of women (30–40%) were grouped into high or moderate classes for both physical and emotional IPV. These women exhibit sustained risk and were exposed to prolonged high or moderate intensity IPV. Though this may be a pattern already established and unrelated to pregnancy, it appears to be present during, and persists following, pregnancy. Given that negative child health outcomes have been linked to IPV exposure in utero as well as during early life, this pattern of sustained exposure may represent an important risk to not only the mental and physical well-being of these mothers, but also to that of their children. Women grouped into high or moderate exposure classes over time are key to identify early through screening conducted during routine antenatal care. The majority of women in South Africa access antenatal care and would benefit from targeted screening and referral, particularly for those exposed to ongoing high or moderate IPV.

Our study identified a second risk group, which emerged across emotional and physical IPV sub-types. The largest class of women for both emotional (70%) and physical IPV (60%) exhibited a pattern of increased probability of exposure during pregnancy relative to the postpartum time-points. This is consistent with some previous longitudinal studies, which found increased IPV exposure during pregnancy compared to postpartum (Mahenge et al., 2016). However, there is not a consensus as other studies have cited a reduction in IPV prevalence during pregnancy, compared to pre-pregnancy prevalence (Fawole et al., 2008; Saltzman et al., 2003). This may link to risk associated with increased financial strain during pregnancy (Bacchus et al., 2006), conflict arising from unwanted pregnancy, substance use (Fanslow et al., 2008) or traditional gender norms (Shamu et al., 2011). Higher prevalence during pregnancy may be linked to relationship status as pregnant women are more likely to be partnered. The majority of women in the current study exhibited higher probabilities of IPV exposure during pregnancy; as mentioned, given that the vast majority of women in South Africa attend antenatal care, this provides a critical opportunity to screen for IPV in those with sustained risk over time as well as in women who have increased risk during pregnancy.

A second focus of this study was to inform understanding of the cycle of abuse. In our LMIC sample of women, childhood sexual, physical and emotional abuse, and physical neglect were found to be associated with membership in high IPV exposure classes. The strength of these associations was high, with two to five-fold increased odds depending on type of childhood maltreatment, for women exposed. Our study confirms and extends previous findings linking sexual maltreatment (Dunkle, Jewkes, Brown, Gray et al., 2004; Dunkle, Jewkes, Brown, Yoshihama et al., 2004; Classen et al., 2005; Daigneault et al., 2009) or physical childhood maltreatment and IPV in adulthood (Bensley, Van Eenwyk, & Wynkoop Simmons, 2003; Richards, Tillyer, & Wright, 2017). However, previous research has not found increased risk for those exposed to emotional abuse (McMahon et al., 2015). Notably, we did not find particular evidence of specificity in terms of transmission of risk from type of childhood exposure to type of adult IPV.

Membership of high IPV classes was more robustly associated with early adversity than moderate class membership, particularly for physical IPV and sexual IPV. However, moderate emotional IPV exposure was significantly associated with most types of childhood adversity. This is potentially significant, as emotional IPV is often overlooked both in research and in public health settings, even though emotional IPV may have serious effects on maternal mental health and is often more prevalent than physical IPV. Further, these findings may indicate an important relationship between intensity of IPV exposure and maternal maltreatment in childhood as the strengths of association increased with membership in higher intensity classes across all sub-types of IPV. Further, given associations between early life adversity and adult exposure to violence, children of women in the high or moderate exposure groups may already be at increased risk for victimisation in adulthood as well as negative health outcomes during childhood.

To further investigate the cycle of abuse across the lifespan, we explored overall patterns for combined IPV sub-types. This approach allowed investigation of differential patterns across IPV sub-types, important given that these are known to co-occur. Expected patterns emerged for three classes (high, moderate and low classes), these patterns were similar to those that emerged when investigating IPV sub-types separately, as described above. However, an interesting additional class emerged with a very high probability of exposure to IPV during pregnancy, which decreased over time. There were signals for this in the LCGA analysis by separate IPV sub-types, where the largest class of women had slightly elevated levels of IPV antenatally. However, in the combined analysis, antenatal probabilities were extremely high for moderate or high levels of IPV (almost 100% for emotional IPV, 80% for physical IPV and 30% for sexual IPV), which decreased to 20%, 30% and 5% respectively by 2 years postpartum. Importantly, this group was 10% of the study sample and so represents a large proportion of pregnant women in the population. This decrease may be due to mothers exiting violent relationships after birth of their child. Alternatively this pattern could represent a high risk for IPV during pregnancy associated with conflict related to financial strain or an unexpected pregnancy which dissipates with time. For these women, though the risk decreases postnatally, there are significant health risks for their child linked to in utero exposure. Importantly, screening during antenatal care provides a critical opportunity to intervene.

In the combined IPV class analysis, similar to the analysis by IPV sub-type, all domains of maternal childhood maltreatment were significantly associated with membership in the high versus low class. However, in looking at the childhood maltreatment patterns predicting membership of moderate vs low combined IPV class, only childhood physical abuse reached significance in our cohort. Where analysis split IPV subtypes for the mothers, all childhood maltreatment groups (with the exception of sexual abuse) was associated with moderate emotional IPV class membership and only the no childhood maltreatment group was associated with moderate physical IPV class membership. This suggests a differential relationship between maternal childhood maltreatment and later emotional IPV, an association which may be lost when IPV sub-types are combined. In combined IPV models, emotional neglect in childhood was associated with membership in the decreasing IPV class, other childhood maltreatment variables were not. This group shows very high probabilities during pregnancy, a critical time for both maternal and child health. Further work is needed to better understand the increased risk for IPV during the antenatal period as well as factors which support the decrease in risk postnatally, patterns that may be due to other personal, community or family-level factors and which may provide useful targets for intervention strategies.

Importantly, poly-victimisation was prevalent in the current study; 38% of women were exposed to two or more types of maltreatment in childhood and 27% were exposed to two or more types of IPV. Further, the combined IPV sub-type LCA analysis, underscores the prevalence of polyvictimisation across IPV sub-type, including the frequency and intensity of exposures, which were largely stable across sub-types. There is a dearth of evidence from LMIC settings, where unique cultural and social factors may impact associations differently than in high-income country settings. This may be particularly relevant in environments such as South Africa, with high rates of child maltreatment as well as IPV exist. High levels of violence in South Africa generally may facilitate a culture where IPV is normalised, thereby increasing the risk of cumulative or long-term exposure as women remain in problematic relationships (Mpondo, Ruiter, van den Borne, & Reddy, 2016; Zembe et al., 2015). The relatively low levels of education and economic power of women in South Africa may further exacerbate the vulnerability of mothers to IPV (Shamu et al., 2011). Given the negative physical and mental health outcomes associated with IPV exposure, for both mothers and children, it is essential that we understand the specific processes via which early adversity confers risk, and consider potential intervention targets and optimal time points to intervene.

Guidelines on care of women exposed to domestic violence in South Africa and the Western Cape are available, however, existing guidelines are not universally adopted and the implementation is often lacking in continuity and poorly coordinated (Joyner & Mash, 2014). More effective procedures are in place for sexual abuse than for physical or emotional IPV which may be more difficult to recognise. There is much work that is required to ensure that women in need are identified and supported in accessing care or resources. Previous studies have found that healthcare providers may resist identifying and managing IPV as a health issue, especially given its complexity and the need for long-term support (Rees, Zweigenthal, & Joyner, 2014). Low levels of IPV identification have been noted, even though women routinely present with physical and mental symptoms indicative of IPV exposure, including injury, anxiety and depression (Joyner & Mash, 2012; Rees et al., 2014). One study found that only 10% of women presenting at primary care facilities while suffering from IPV were identified as such (Joyner & Mash, 2014). IPV is a complex issue, requiring a multi-sectorial approach to address risk factors and provide support to women affected. Nonetheless, the majority of pregnant women in South Africa present to primary health facilities for antenatal care, offering a critical opportunity for identification and referral. The present study offers insight into patterns of IPV exposure during and following pregnancy as well as key risk factors, which may be possible to adapt for screening purposes in primary healthcare facilities. In low resource settings, it may be necessary to triage resources to identify the most critical cases. The longitudinal profiles described in the current research indicate that many women are at sustained risk of exposure during and following pregnancy, with a key group emerging that is at increased antenatal risk. This highlights the need for a universal screening programme at the primary healthcare level, with a priority focus given to pregnant mothers.

### 5.1 Strengths & limitations

Although inclusion criteria for the DCHS were broad to ensure generalisability, recruitment was done during antenatal care visits; mothers who did not present for antenatal care were therefore not captured, which may have resulted in the highest risk mothers being underrepresented in the sample. There may be some bias present due to the timing of assessments, which had shorter intervals between the 1^st^/2^nd^ and 2^nd^/3^rd^ study visits compared to subsequent 6-monthly visits. This may have affected the patterns of IPV reported across time. In addition, 205 mothers who were enrolled in the study were not included in the analysis due to incomplete data, although there was no evidence that these excluded mothers differed from the wider sample on key risk factors. Finally, due to collinearity of types of childhood maltreatment, each was included in a separate model, we were therefore unable to determine additive risk for a particular type of childhood maltreatment.

Despite these limitations, the current study is among the first to investigate associations between sub-types of maternal maltreatment in childhood and adult IPV exposure in a LMIC. A key strength includes longitudinal assessment of IPV exposure. Our findings corroborate previous research and build upon it by investigating sub-types of IPV during pregnancy including investigating intensity of exposure. Two key groups emerged in our research – one at sustained risk of high or moderate IPV during pregnancy and postpartum; the second exhibited increased probability of IPV exposure during pregnancy compared to postpartum. The majority of women in South Africa access antenatal care and both of these groups would benefit from targeted screening and referral, particularly during this sensitive period for both maternal and child health.

### 5.2 Data sharing

Collaborations for the analysis of data are welcome; the DCHS has a large and active group of investigators and postgraduate students and many have successfully partnered with students or researchers from other institutions. Researchers who are interested in datasets or collaborations can find more information on our website [http://www.paediatrics.uct.ac.za/scah/dclhs].

## Funding

The study was funded by the Bill and Melinda Gates Foundation [OPP 1017641]. Additional support for HJZ and DJS by the MRC of South Africa. Additional aspects of the work reported here are supported by the South African NRF and MRC, by an Academy of Medical Sciences Newton Advanced Fellowship (NAF002\1001), funded by the UK Government’s Newton Fund and by the US Brain and Behaviour Foundation Independent Investigator grant (24467). WB is supported by the SAMRC National Health Scholars programme. AF is supported by a personal fellowship from the UK MRC [grant number MR/M009351/1] and works in a Unit that receives core funding from UK MRC [grant number MC_UU_12013/5].

## References

[bib0005] Abrahams N., Jewkes R. (2005). Effects of South African men’s having witnessed abuse of their mothers during childhood on their levels of violence in adulthood. American Journal of Public Health.

[bib0010] Abrahams N., Matthews S., Jewkes R., Martin L., Lombard C. (2012). Every eight hours: Intimate femicide in South African 10 years later!.

[bib0015] Abramsky T., Watts C.H., Garcia-Moreno C., Devries K., Kiss L., Ellsberg M., Jansen H., Heise L. (2011). What factors are associated with recent intimate partner violence? Findings from the WHO multi-country study on women’s health and domestic violence. BMC Public Health.

[bib0020] Asparouhov T., Muthén B. (2014). Auxiliary variables in mixture modeling: A 3-Step approach using mplus. https://www.statmodel.com/download/webnotes/webnote15.pdf.

[bib0025] Bacchus L., Mezey G., Bewley S. (2006). A qualitative exploration of the nature of domestic violence in pregnancy. Violence Against Women.

[bib0030] Bensley L., Van Eenwyk J., Wynkoop Simmons K. (2003). Childhood family violence history and women’s risk for intimate partner violence and poor health. American Journal of Preventative Medicine.

[bib0035] Bernstein D.P., Fink L., Handelsman L., Foote J., Lovejoy M., Wenzel K., Sapareto E., Ruggiero J. (1994). Initial reliability and validity of a new retrospective measure of child abuse and neglect. The American Journal of Psychiatry.

[bib0040] Bernstein M., Phillips T., Zerbe A., McIntyre J.A., Brittain K., Petro G., Abrams E.J., Myer L. (2016). Intimate partner violence experienced by HIV-infected pregnant women in South Africa: A cross-sectional study. BMJ Open.

[bib0045] Bronte-Tinkew J., Zaslow M., Capps R., Horowitz A., McNamara M. (2007). Food insecurity works through depression, parenting, and infant feeding to influence overweight and health in toddlers. The Journal of Nutrition.

[bib0050] Burton P., Artz L W.C. (2015). The optimus study on child abuse, violence and neglect in South Africa..

[bib0055] Campbell J.C. (2002). Health consequences of intimate partner violence. Lancet.

[bib0060] Classen C.C., Palesh O.G., Aggarwal R. (2005). Sexual revictimization: A review of the empirical literature. Trauma, Violence & Abuse.

[bib0065] Daigneault I., Hebert M., McDuff P. (2009). Men’s and women’s childhood sexual abuse and victimization in adult partner relationships: A study of risk factors. Child Abuse & Neglect.

[bib0070] Dunkle K.L., Jewkes R.K., Brown H.C., Gray G.E., McIntryre J.A., Harlow S.D. (2004). Gender-based violence, relationship power, and risk of HIV infection in women attending antenatal clinics in South Africa. Lancet.

[bib0075] Dunkle K.L., Jewkes R.K., Brown H.C., Yoshihama M., Gray G.E., McIntyre J.A. (2004). Prevalence and patterns of gender-based violence and revictimization among women attending antenatal clinics in Soweto, South Africa. American Journal of Epidemiology.

[bib0080] Duvvury N., Callan A., Carney P., Raghavendra S. (2013). Intimate partner violence: Economic costs and implications for growth and development. https://openknowledge.worldbank.org/handle/10986/16697.

[bib0085] Fanslow J., Silva M., Robinson E., Whitehead A. (2008). Violence during pregnancy: Associations with pregnancy intendedness, pregnancy-related care, and alcohol and tobacco use among a representative sample of New Zealand women. The Australian & New Zealand Journal of Obstetrics & Gynaecology.

[bib0090] Fawole A.O., Hunyinbo K.I., Fawole O.I. (2008). Prevalence of violence against pregnant women in Abeokuta, Nigeria. The Australian & New Zealand Journal of Obstetrics & Gynaecology.

[bib0095] Feingold A., Tiberio S.S., Capaldi D.M. (2014). New approaches for examining associations with latent categorical variables: Applications to substance abuse and aggression. Psychology of Addictive Behaviors.

[bib0100] Galatzer-Levy I.R. (2015). Applications of latent growth mixture modeling and allied methods to posttraumatic stress response data. European Journal of Psychotraumatology.

[bib0105] Gilbert A.L., Bauer N.S., Carroll A.E., Downs S.M. (2013). Child exposure to parental violence and psychological distress associated with delayed milestones. Pediatrics.

[bib0110] Groves A., Kagee A., Maman S., Moodley D., Rouse P. (2012). Associations between intimate partner violence and emotional distress among pregnant women in Durban, South Africa. Journal of Interpersonal Violence.

[bib0115] Groves A.K., Moodley D., McNaughton-Reyes L., Martin S.L., Foshee V., Maman S. (2015). Prevalence and rates of intimate partner violence among South African women during pregnancy and the postpartum period. Maternal and Child Health Journal.

[bib0120] Gustafsson H.C., Coffman J.L., Cox M.J. (2015). Intimate partner violence, maternal sensitive parenting behaviors, and children’s executive functioning. Psychology of Violence.

[bib0125] Huth-Bocks A.C., Levendosky A.A., Bogat G.A. (2002). The effects of domestic violence during pregnancy on maternal and infant health. Violence and Victims.

[bib0130] Jewkes R. (2002). Intimate partner violence: Causes and prevention. Lancet..

[bib0135] Jewkes R., Nduna M., Levin J., Jama N., Dunkle K., Puren A. (2008). Impact of stepping stones on incidence of HIV and HSV-2 and sexual behaviour in rural South Africa: Cluster randomised controlled trial. BMJ.

[bib0140] Jewkes R., Penn-Kekana L., Levin J., Ratsaka M., Schrieber M. (2001). Prevalence of emotional, physical and sexual abuse of women in three South African provinces. South African Medical Journal.

[bib0145] Jewkes R., Dunkle K., Koss M.P., Levin J.B., Nduna M., Jama N. (2006). Rape perpetration by young, rural South African men: Prevalence, patterns and risk factors. Social Science & Medicine.

[bib0150] Jewkes R., Dunkle K., Nduna M., Levin J., Jama N., Khuzwayo N., Koss M., Puren A., Duvvury N. (2006). Factors associated with HIV sero-status in young rural South African women: Connections between intimate partner violence and HIV. International Journal of Epidemiology.

[bib0155] Joyner K., Mash B. (2012). Recognizing intimate partner violence in primary care: Western Cape, South Africa. PloS One.

[bib0160] Joyner K., Mash B. (2014). Quality of care for intimate partner violence in South African primary care: A qualitative study. Violence and Victims.

[bib0165] Li Y., Marshall C.M., Rees H.C., Nunez A., Ezeanolue E.E., Ehiri J.E. (2014). Intimate partner violence and HIV infection among women: A systematic review and meta-analysis. Journal of the International AIDS Society.

[bib0170] Ludermir A.B., Lewis G., Valongueiro S.A., de Araujo T.V., Araya R. (2010). Violence against women by their intimate partner during pregnancy and postnatal depression: A prospective cohort study. Lancet.

[bib0175] Mahenge B., Stöckl H., Abubakari A., Mbwambo J., Jahn A. (2016). Physical, sexual, emotional and economic intimate partner violence and controlling behaviors during pregnancy and postpartum among women in Dar es Salaam, Tanzania. PloS One.

[bib0180] Maman S., Mbwambo J.K., Hogan N.M., Kilonzo G.P., Campbell J.C., Weiss E. (2002). HIV-positive women report more lifetime partner violence: findings from a voluntary counseling and testing clinic in Dar es Salaam, Tanzania. American Journal of Public Health.

[bib0185] Mathews S., Abrahams N., Martin L.J., Vetten L., van der Merwe L., Jewkes R. (2004). Every six hours a woman is killed by her intimate partner: A national study of female homicide in South Africa.

[bib0190] McKinney C.M., Caetano R., Ramisetty-Mikler S., Nelson S. (2009). Childhood family violence and perpetration and victimization of intimate partner violence: Findings from a national population-based study of couples. Annals of Epidemiology.

[bib0195] McMahon K., Hoertel N., Wall M.M., Okuda M., Limosin F., Blanco C. (2015). Childhood maltreatment and risk of intimate partner violence: A national study. Journal of Psychiatric Research.

[bib0200] Meinck F., Cluver L.D., Boyes M.E. (2015). Household illness, poverty and physical and emotional child abuse victimisation: findings from South Africa’s first prospective cohort study. BMC Public Health.

[bib0205] Meyer S.R., Yu G., Hermosilla S., Stark L. (2017). Latent class analysis of violence against adolescents and psychosocial outcomes in refugee settings in Uganda and Rwanda. Global Mental Health (Cambridge Core).

[bib0210] Mpondo F., Ruiter R.A., van den Borne B., Reddy P.S. (2016). Intimate partner violence and its association with self-determination needs and gender-power constructs among rural South African Women. Journal of Interpersonal Violence.

[bib0215] Muthen B., Muthen L.K. (2000). Integrating person-centered and variable-centered analyses: Growth mixture modeling with latent trajectory classes. Alcoholism, Clinical and Experimental Research.

[bib0220] Myer L., Stein D.J., Grimsrud A., Seedat S., Williams D.R. (2008). Social determinants of psychological distress in a nationally-representative sample of South African adults. Social Science & Medicine.

[bib0225] Olson S.L., Choe D.E., Sameroff A.J. (2017). Trajectories of child externalizing problems between ages 3 and 10 years: Contributions of children’s early effortful control, theory of mind, and parenting experiences. Development and Psychopathology.

[bib0230] Rees K., Zweigenthal V., Joyner K. (2014). Implementing intimate partner violence care in a rural sub-district of South Africa: A qualitative evaluation. Global Health Action.

[bib0235] Richards T.N., Tillyer M.S., Wright E.M. (2017). Intimate partner violence and the overlap of perpetration and victimization: Considering the influence of physical, sexual, and emotional abuse in childhood. Child Abuse & Neglect.

[bib0240] Saltzman L.E., Johnson C.H., Gilbert B.C., Goodwin M.M. (2003). Physical abuse around the time of pregnancy: An examination of prevalence and risk factors in 16 states. Maternal and Child Health Journal.

[bib0245] Shamu S., Abrahams N., Temmerman M., Musekiwa A., Zarowsky C. (2011). A systematic review of African studies on intimate partner violence against pregnant women: Prevalence and risk factors. PloS One.

[bib0250] Shamu S., Zarowsky C., Roelens K., Temmerman M., Abrahams N. (2016). High-frequency intimate partner violence during pregnancy, postnatal depression and suicidal tendencies in Harare, Zimbabwe. General Hospital Psychiatry.

[bib0255] Solomon M., Iosif A.M., Reinhardt V.P., Libero L.E., Nordahl C.W., Ozonoff S., Rogers S.J., Amaral D.G. (2018). What will my child’s future hold? Phenotypes of intellectual development in 2–8-year-olds with autism spectrum disorder. Autism Research.

[bib0260] Stein D.J., Koen N., Donald K.A., Adnams C.M., Koopowitz S., Lund C., Marais A., Myers B., Roos A., Sorsdahl K., Stern M., Tomlinson M., van der Westhuizen C., Vythilingum B., Myer L., Barnett W., Brittain K., Zar H.J. (2015). Investigating the psychosocial determinants of child health in Africa: The drakenstein child health study. Journal of Neuroscience Methods.

[bib0265] Tiwari A., Chan K., Fong D., Leung W., Brownridge D., Lam H., Wong B., Lam C., Chau F., Chan A., Cheung K., Ho P. (2008). The impact of psychological abuse by an intimate partner on the mental health of pregnant women. BJOG an International Journal of Obstetrics and Gynaecology.

[bib0270] Udo I.E., Sharps P., Bronner Y., Hossain M.B. (2016). Maternal intimate partner violence: Relationships with language and neurological development of infants and toddlers. Maternal and Child Health Journal.

[bib0275] Valladares E., Ellsberg M., Pena R., Hogberg U., Persson L.A. (2002). Physical partner abuse during pregnancy: A risk factor for low birth weight in Nicaragua. Obstetrics and Gynecology.

[bib0280] Xu X., Zhu F., O’Campo P., Koenig M.A., Mock V., Campbell J. (2005). Prevalence of and risk factors for intimate partner violence in China. American Journal of Public Health.

[bib0285] Yan E., Karatzias T. (2016). Childhood abuse and current intimate partner violence: A population study in Hong Kong. Journal of Interpersonal Violence.

[bib0290] Zar H.J., Barnett W., Myer L., Stein D.J., Nicol M.P. (2015). Investigating the early-life determinants of illness in Africa: The drakenstein child health study. Thorax.

[bib0295] Zembe Y.Z., Townsend L., Thorson A., Silberschmidt M., Ekstrom A.M. (2015). Intimate partner violence, relationship power inequity and the role of sexual and social risk factors in the production of violence among young women who have multiple sexual partners in a Peri-Urban Setting in South Africa. PloS One.

