# Supplementary material for: Maltreatment in childhood and intimate partner violence: A latent class growth analysis in a South African pregnancy cohort
Source: Child Abuse Negl. 2018 Dec;86:336–48. doi: 10.1016/j.chiabu.2018.08.020 (PMC6297167; doi:10.1016/j.chiabu.2018.08.020)
Supplement: Supplementary file 1 [file mmc1.docx]

**Supplemental Table 1.** Sensitivity analysis of key study variables

|  |  | | **Included sample (n=832)** | **Excluded sample (n=205)** | **Total sample, n** | | **p-value** |
| --- | --- | --- | --- | --- | --- | --- | --- |
| Demographic variables^a^ | | |  |  |  | |  |
|  | TC Newman | | 381 (47) | 123 (60) | 1037 | | 0.074 |
| Maternal education | | |  |  |  | |  |
|  | Some secondary | | 531 (64) | 114 (57) | 1030 | | 0.087 |
|  | Completed secondary | | 301 (36) | 85 (43 |  | |  |
| Maternal birthplace | | |  |  |  | |  |
|  | Outside Paarl | | 302 (36) | 83 (42) | 1030 | | 0.156 |
|  | Paarl | | 530 (64) | 116 (58) |  | |  |
| Maternal Employment | | |  |  |  | |  |
|  | Unemployed | | 628 ( 75) | 133 (68) | 1026 | | 0.058 |
|  | Working | | 204 (25) | 62 (32) |  | |  |
| Partnership status | | |  |  |  | |  |
|  | Single | | 496 (60) | 115 (57) |  | |  |
|  | Married/marriage-like | | 336 (40) | 85 (43) | 1031 | | 0.572 |
| Income |  | |  |  |  | |  |
|  | <R1,000/mo | | 327 (39) | 68 (33) | 1037 | | 0.118 |
|  | >R1,000/mo | | 506 (61) | 136 (67) |  | |  |
| Maternal age | | 26.2 (22.1, 30.9) | | 24.7 (21.3, 29.9) | | 1037 | 0.053 |
| Childhood Maltreatment (above threshold) | | |  |  |  | |  |
|  | Physical Neglect | | 279 (34) | 83 (41) | 1037 | | 0.061 |
|  | Emotional Abuse | | 263 (32) | 53 (25) | 1037 | | 0.100 |
|  | Emotional Neglect | | 230 (28) | 70 (34) | 1037 | | 0.060 |
|  | Physical Abuse | | 189 (23) | 39 (19) | 1037 | | 0.273 |
|  | Sexual Abuse | | 137 (16) | 33 (16) | 1037 | | 0.931 |
| Intimate Partner Violence (above threshold) | | | |  |  | |  |
|  | Emotional | | 205 (25) | 45 (22) | 1035 | | 0.121 |
|  | Physical | | 154 (19) | 32 (16) | 1035 | | 0.391 |
|  | Sexual | | 50 (6) | 11 (5) | 1035 | | 0.427 |

*^a^demographic variables were collected antenatally at 28-32weeks’ gestation*

**Supplemental Table 2.** Intimate partner prevalence by sub-type and visit time point^a^

*^a^Descriptive data for categories of IPV exposure by visit time point (these categories were used to generate latent classes).*

|  | Antenatal  n (%) | | 10 Weeks  n (%) | 6 Months  n (%) | 12 Months  n (%) | 18 Months  n (%) | 24 Months  n (%) |
| --- | --- | --- | --- | --- | --- | --- | --- |
| *Number of mothers* | | *832* | *582* | *560* | *663* | *581* | *570* |
| Emotional IPV |  | |  |  |  |  |  |
| No IPV | 557 (67) | | 445 (76) | 401 (72) | 497 (75) | 458 (79) | 451 (79) |
| Isolated/Low | 70 (8) | | 32 (6) | 35 (6) | 30 (5) | 26 (5) | 28 (5) |
| Moderate | 128 (15) | | 63 (11) | 72 (13) | 77 (12) | 50 (9) | 42 (7) |
| High | 77 (9) | | 42 (7) | 52 (9) | 59 (9) | 47 (8) | 49 (9) |
| Physical IPV |  | |  |  |  |  |  |
| No IPV | 586 (70) | | 447 (77) | 421 (75) | 510 (77) | 463 (80) | 454 (80) |
| Isolated/Low | 92 (11) | | 40 (8) | 44 (8) | 35 (5) | 31 (5) | 28 (5) |
| Moderate | 102 (12) | | 59 (10) | 57 (10) | 76 (12) | 51 (9) | 50 (9) |
| High | 52 (6) | | 36 (6) | 38 (7) | 42 (6) | 36 (6) | 38 (7) |
| Sexual IPV |  | |  |  |  |  |  |
| No IPV | 760 (91) | | 544 (94) | 523 (93) | 627 (95) | 553 (95) | 539 (95) |
| Isolated/Low | 22 (3) | | 11 (2) | 10 (2) | 9 (1) | 8 (1) | 4 (1) |
| Moderate | 33 (4) | | 22 (4) | 21(4) | 17 (3) | 15 (3) | 18 (3) |
| High | 17 (2) | | 5 (1) | 6 (1) | 10 (2) | 5 (1) | 9 (2) |
